# No need to touch this: Bimanual haptic slant adaptation does not require touch

**DOI:** 10.1371/journal.pone.0236824

**Published:** 2020-07-31

**Authors:** Catharina Glowania, Myrthe A. Plaisier, Marc O. Ernst, Loes C. J. Van Dam

**Affiliations:** 1 Cognitive Neuroscience Department and Cognitive Interaction Technology—Center of Excellence, Bielefeld University, Bielefeld, Germany; 2 Department of Mechanical Engineering, Dynamics & Control group, Eindhoven University of Technology, Eindhoven, The Netherlands; 3 Applied Cognitive Psychology, Institute for Psychology, Ulm University, Ulm, Germany; 4 Department of Psychology, University of Essex, Colchester, United Kingdom; Birkbeck University of London, UNITED KINGDOM

## Abstract

In our daily life, we often interact with objects using both hands raising the question the question to what extent information between the hands is shared. It has, for instance, been shown that curvature adaptation aftereffects can transfer from the adapted hand to the non-adapted hand. However, this transfer only occurred for dynamic exploration, e.g. by moving a single finger over a surface, but not for static exploration when keeping static contact with the surface and combining the information from different parts of the hand. This raises the question to what extent adaptation to object shape is shared between the hands when both hands are used in static fashion simultaneously and the object shape estimates require information from both hands. Here we addressed this question in three experiments using a slant adaptation paradigm. In Experiment 1 we investigated whether an aftereffect of static bimanual adaptation occurs at all and whether it transfers to conditions in which one hand was moving. In Experiment 2 participants adapted either to a felt slanted surface or simply be holding their hands in mid-air at similar positions, to investigate to what extent the effects of static bimanual adaptation are posture-based rather than object based. Experiment 3 further explored the idea that bimanual adaptation is largely posture based. We found that bimanual adaptation using static touch did lead to aftereffects when using the same static exploration mode for testing. However, the aftereffect did not transfer to any exploration mode that included a dynamic component. Moreover, we found similar aftereffects both with and without a haptic surface. Thus, we conclude that static bimanual adaptation is of proprioceptive nature and does not occur at the level at which the object is represented.

## Introduction

In our daily life we often use both of our hands in many haptic tasks, such as doing the dishes, typing text using a computer keyboard or playing a musical instrument. When performing such tasks, the movements of the two hands are relatively independent, at least at a mechanical level. That is, activating the muscles of one arm/hand does not lead to a movement of the other. For instance, when playing the guitar one hand frets the chords while the other hand plucks the guitar strings without the one task interfering mechanically with the other because each hand is controlled by a separate set of muscles. However, for performing such bimanual tasks the two hands do of course still need to be coordinated by the Central Nervous System (CNS) leading to the question to what extent and at what stages sensory information is combined. Even when haptically exploring objects we often use both of our hands in a coordinated fashion. [1] investigated object exploration with both one and two hands and showed that the modes of exploration used to obtain information about the object properties are very specialized and coordinated across the hands. That is, the exploratory actions we make are very specific to the object property we want to explore. For instance, we dynamically slide with the fingers over a surface for texture information but we statically hold an object in our hands to estimate its weight; and when exploring the shape of an object, we often hold the object with one hand and move with the other over its surface. However, object shape information can be obtained in multiple ways: we can do so by statically touching the object with a large portion of our hand(s) (static exploration) or by dynamically moving with our finger(s) over its surface (dynamic exploration). Moreover, we can explore object shape using either one or both hands.

It is important to note however, that research on haptic shape perception has often involved paradigms that use only one hand instead of two. This is particularly the case for haptic shape adaptation studies in which participants are exposed to a curved or slanted surface for a prolonged period of time. Afterwards a flat/level surface is perceived as curved or slanted in the opposite direction (the haptic adaptation aftereffect). So far, haptic shape adaptation studies focused on conditions in which only a single hand was adapted, be it by sliding over a surface with one finger [2, 3], touching the surface with the whole hand [4, 5] or multiple fingers [3], touching a small part of a surface with the fingertip [6] or rubbing thumb and fingers along the sides of a bar [7]. In the present study, we will instead investigate bimanual haptic adaptation by using the index fingers of both hands simultaneously to make a perceptual judgment, and the potential transfer to other exploration modes.

Note that in the mentioned examples, often one hand or even one finger was sufficient to obtain the required information to estimate the surface shape. Using two hands instead of one in these cases would mean that each hand provides a separate estimate of object shape. That is, the two hands would provide redundant information. However, for large curvatures or slanted surfaces one finger, if used in a static fashion, does not provide very meaningful information of such global shapes. In such cases, one finger alone samples too small a portion of the surface to provide a very reliable estimate of the curvature or slant [8, 9]. This means that for global shape estimation by static touch, at least one additional finger is needed, be it from the same or opposite hand. In this case, the information provided by the additional finger is no longer redundant; instead, this information is necessary to estimate the shape. The difference in position between the fingers when touching the object (e.g. due to the difference in height at which the object is touched) would be informative about the object’s shape [10].

Previous studies have focused on shape perception using multiple fingers from one hand (e.g. [2, 3]) and found that adaptation largely depends on the posture of the hand. However, whereas two fingers from the same hand are mechanically coupled to some extent (i.e. they partially use the same set of muscles), the fingers from the opposite hands share no mechanical coupling, in e.g. muscles and skin, and thus do not directly share any low-level receptors at which adaptation can occur. Therefore, any bilateral control or coupling of sensory information between the hands has to take place in the CNS, e.g. through bilateral tactile receptive fields in the primary somatosensory cortex [11–14] which is another potential stage at which adaptation may occur. However, it is unclear which of these stages would contribute to perceptual shape adaptation aftereffects in the case of static bimanual exploration. In order to investigate whether shape adaptation aftereffects still occur in this case, the present study will particularly focus on the situation when two fingers from our two separate hands are used for adaptation (we will use both the index fingers of the left and right hand). In order to perceive the global shape by using the left and right index finger, the two hands need to share their position information to create a combined percept. If we find adaptation aftereffects for this mode of exploration, the intuitive conclusion seems to be that adaptation occurs at this bimanual position sharing stage. However, as will become evident our results rather point towards static bimanual adaptation still being posture based and at the level of the individual hands.

We conducted three experiments. Experiment 1 and 2 tested contrasting predictions of non-redundant bimanual slant adaptation being posture based or occurring at the level of the bimanual surface representation. Experiment 1 tested whether non-redundant bimanual adaptation transfers to conditions that include a dynamic exploration component and Experiment 2 investigated whether or not a surface is needed to be felt for haptic slant adaptation to occur. As will become clear the results of both these experiments indicated that haptic adaptation was driven by posture, rather than adaptation occurring at the processing level at which the surface is represented. This would mean that bimanual adaptation aftereffects are based on the comparison of two individually adapted hands by the brain [15, 16], and thus, adapting only one hand might be sufficient to show adaptation aftereffects. This was confirmed in a third and last experiment in which only one hand was adapted to a position in space and clear aftereffects of adaptation were found.

## Experiment 1

In Experiment 1 we tested whether static bimanual slant adaptation occurs when the information of the two hands is non-redundant (i.e. the slant estimate cannot be obtained using one hand alone). If so, it would seem intuitive that such adaptation occurs at the level at which the information of the two hands is shared. Evidence for information sharing between the hands for shape perception was previously found for dynamic unimanual exploration by studies that investigated transfer of haptic adaptation between the hands. In a study by Van der Horst et al. [2] participants adapted dynamically to haptic curvature (i.e. they moved a single finger back and forth over the surface) and showed transfer of the aftereffects to the fingers of the opposite hand, which were never directly involved in the adaptation process. Van der Horst and colleagues concluded that the adaptation occurred at a level at which the dynamic information of the two hands is shared. The same was found for virtual surfaces for which adaptation to curvature using a dynamic exploration mode also transferred intermanually [17]. However, for static contact of the surface the intermanual transfer effects were much reduced [6] or even absent [5], suggesting that static touch adaptation might be more specific to the hand used during adaptation. In other words, for static unimanual exploration the literature points towards a more receptor-based adaptation. This suggests that information sharing between the hands may depend on the mode of exploration.

The present case of non-redundant bimanual static adaptation to shape however naturally requires the sharing of information across the hands and therefore may be occurring at a level that generally couples the information from the two hands regardless of exploration. A previous study by Dupin et al. [18], for instance, showed that the kinaesthetic information coming from one hand and tactile information coming from the other hand can be combined in the brain to form a single percept of object shape. If indeed the adaptation occurs at such a general bimanual coupling level at which information of the two hands is available, one could expect adaptation to transfer to conditions with a dynamic component (see e.g. [2, 6]). However, in line with adaptation transfer studies finding different results in static and dynamic conditions, a recent study found that when using the same hand, aftereffects do not transfer between static and dynamic exploration modes [3]. This suggests very distinctive processing pathways for these separate modes of exploration. Furthermore, it is known that the primary and secondary nerve endings in the muscle spindles respond to either position as well as movement or to position alone, respectively. Therefore, it is also possible that any static bimanual adaptation is exploration mode specific and thus does not occur at a higher level at which bimanual dynamic information is represented. Thus, if bimanual adaptation is exploration mode specific, this would point to adaptation occurring at a less general and thus likely a more pre-CNS stage involving skin and muscle receptors or the very early processing thereof in the CNS.

In short, the purpose of Experiment 1 was twofold: First we investigated whether static bimanual slant adaptation occurs when the information of the two hands is non-redundant. In order to do so participants adapted to a slanted surface by touching the surface with their two index fingers statically. The adaptation aftereffect was measured using this same static bimanual exploration mode in the test phase. Second, to test whether static bimanual adaptation is exploration mode specific as well as to gain insights into the level at which bimanual static adaptation may occur, Experiment 1 included transfer conditions that had a dynamic exploration component (either moving one finger over the surface or moving one finger and keeping static contact with the other).

### Material and methods experiment 1

#### Participants

Informed consent was acquired prior to participation and participants were treated in accordance with the Declaration of Helsinki. Ethical approval was obtained from the Bielefeld University ethics committee. Thirteen people (including the authors CG and LD) volunteered to participate in the experiment (11 female, all participants were right-handed upon self-report, age range: 19–38). Note that this number of participants is generally sufficient for haptic adaptation studies, since effect sizes of haptic adaptation aftereffects tend to be relatively large (e.g. [2, 4, 5, 8] used participant numbers ranging between 2 and 8 for separate experiments). The students received financial compensation (6€/h) for their participation. None of the participants reported any somatosensory deficits.

#### Setup

**Fig 1:**
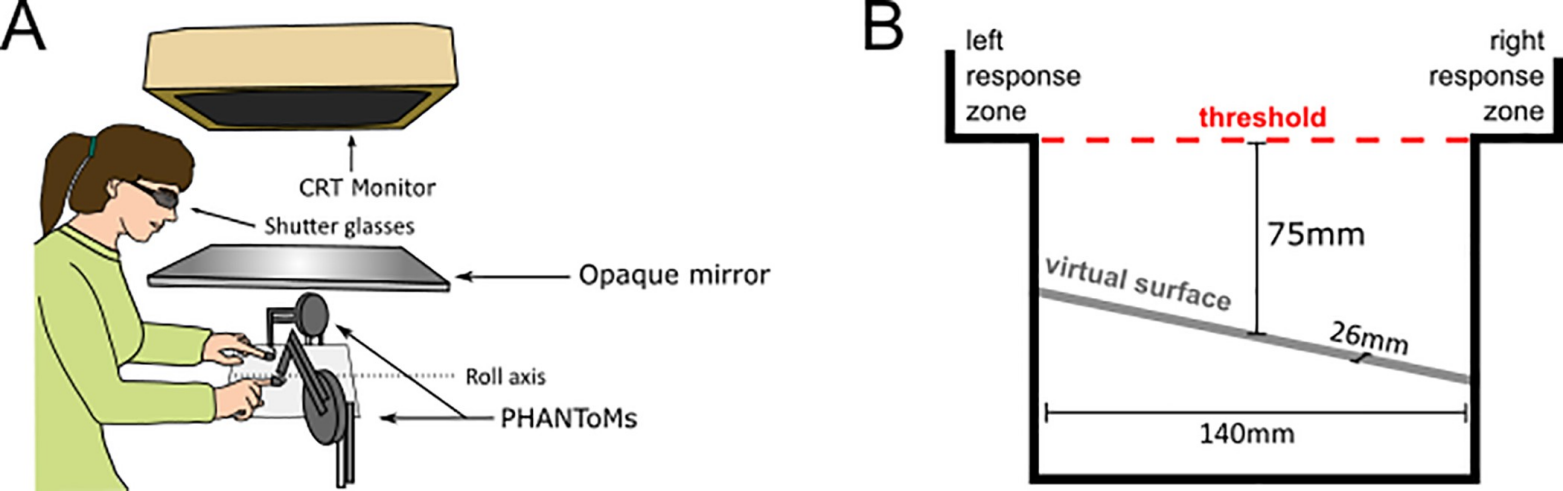
Experimental and virtual setup. A: Experimental Setup. The participant was seated in front of a visuo-haptic workbench consisting of a CRT-monitor, an opaque mirror and two PHANToM force feedback devices which were attached to the participants left and right index fingers; B: Virtual Setup. workspace box that contains the virtual surface (depth 26 mm) as well as response zones at the top left and right of the box; The red dashed line indicates the threshold that participants had to cross with both index fingers in order to start the trial.

The participants were seated behind a haptic workbench on which two PHANToM force-feedback devices (PHANToM premium 1.5, SensAble Technologies, Inc. Woburn, MA) were mounted–with their body midline aligned with the centre of the bench. On each side of the workbench one PHANToM force-feedback device was placed. Participants placed their right and left index fingers into thimble-like holders, attached to each PHANToM (see Fig 1A). The PHANToMs were used to render virtual slanted surfaces and the haptic rendering could be switched on and off independently for each finger. Thus, haptic information could be displayed to both fingers simultaneously or to only one of the fingers individually. Furthermore, the PHANToMs were used to record the participant’s movement trajectories during exploration to verify adherence to the task. For the current experiment, the system was setup to record the finger positions with a sampling rate of 47Hz. To inform the participants about the next trial, a CRT monitor (Sony CPD G500/G500J, Sony Europe Limited, Weybridge, UK; 140 Hz) was used.

#### Stimuli & procedure

For adaptation, we always used a static bimanual exploration mode whereas for the test trials there were three exploration modes: Static Bimanual (adapted condition), Dynamic Unimanual (transfer condition 1) and Mixed Bimanual (transfer condition 2). In the Static Bimanual mode, participants kept static contact with the surface using the index fingers of the left and right hands. In the Dynamic Unimanual condition, the participants moved their right index finger across the surface in an area spanning 140 mm left to right, centred at body midline, in order to explore the slanted plane. In this condition, the haptic rendering for the left index finger was switched off and thus no haptic information was provided to that finger. In the Mixed Bimanual condition, the surface was again rendered for both the right and left index fingers. In this case, however, participants kept static contact with the left index finger on the left side of the slanted surface and moved across the surface with the right index finger. The Mixed Bimanual condition tested the influence of the bimanual adaptation on an exploration mode that contains both a static and a dynamic component. To avoid the dynamic finger from making contact with the static finger as much as possible, the participants were told to place the static left index finger close to the left end of the surface and to make movements that do not interfere with the static finger. In order to prevent the participants from moving diagonally over the surface in the Dynamic Unimanual and Mixed Bimanual conditions and thus creating the impression of a less slanted surface, we limited the space in the z-direction (depth) by flanking each side of the slant with hard vertical surfaces. The so restricted area for exploration was limited to 26mm in depth, while keeping the entire width of 140 mm.

Before the trial started, participants were informed about which exploration mode to use for the upcoming trial. For this purpose, colour cues were used (red, green and blue), which covered the full range of the screen. A red screen indicated that participants should use the Static Bimanual exploration mode; A green screen was used for the Dynamic Unimanual mode and a blue screen was used for the Mixed Bimanual mode. To make sure the participants used the colour cues adequately, each participant practiced using the correct exploration modes corresponding to the colour cues before the start of the experiment. Moreover, during the experiment, the participant’s finger positions were recorded using the PHANToMs to be able to verify whether the participants adhered to the cues.

In order to start a trial, participants first lifted their fingers above a programmed threshold of 75 mm above the height at which the surface would be rendered. The moment they passed this threshold the colour cue disappeared, and no visual information was provided. Next participants lowered their fingers until they reached the surface and explored the surface for 1s using the exploration mode indicated by the colour cue. The exploration time started as soon as one finger touched the surface and after 1s the surface disappeared. The participants' task was to indicate the slant of the surface by judging which side of the surface felt higher: left or right. Participants provided their response by moving their index finger into the corresponding “response zone” located at the top left and right of the programmed PHANToM workspace (see Fig 1B). Note that also while responding the participants could not see anything on the screen or their finger positions to prevent any interaction from visual cues. The left response zone indicated that the left side was perceived to be higher and vice versa for the right response zone. After providing their response, the exploration mode colour cue for the next trial was shown.

In order to determine the Point of Subjective Equality (PSE)–the point at which the participant perceived the surface as horizontal–we used an adaptive 1-up/1-down staircase procedure (for further information see [19] or [20]). The step size between trials started with 8deg. After two reversals in the responses, the step size was decreased to 4deg. and after another two reversals to 2deg. After 12 reversals, the staircase was terminated.

To measure the effect of slant adaptation we used a pre- versus post-test procedure. In the pre-test as well as in the post-test phases, there were two staircases for each exploration mode. To control for possible hysteresis effects within the staircase procedure one staircase started with a positive angle (+20 deg, right side higher) and the other with a negative angle (-20 deg, left side higher). Hence, 6 staircases were used for each phase (3 exploration modes x 2 staircases) and the trials for these staircases were presented in a randomly interleaved fashion. After all staircases for the pre-test were finished, a message on the screen told the participant to take a break to prevent fatigue from influencing the results. After the break, participants were presented with the adaptation stimulus (surface slant of ±10 deg) for 30s. The direction of adaptation surface slant (to the left or right) was counterbalanced across participants. A colour cue on the screen, like the ones used for test-trials, informed the participant about the exploration mode to use during adaptation. For adaptation, it was always the cue for Static Bimanual exploration. During adaptation participants were not asked to decide which side felt higher. After adaptation, the post-test started. Again, the trials for the 6 staircases were randomly intermixed. However, in the post-test phase, each trial was preceded by 4s top-up adaptation. This means that before the actual trial, the adaptation stimulus was presented for 4s to prevent de-adaptation over time. The top-up adaptation interval was again preceded by the red colour cue, instructing the participant to use the Static Bimanual exploration mode. After the top-up adaptation interval, a second colour cue indicated which exploration mode to use on the upcoming test-trial.

#### Analysis

To calculate the PSEs for each condition we pooled the data from the two staircases (i.e. the staircase starting with a negative slant and the one starting with a positive slant) for each condition in the pre/post-test stage and fitted psychometric curves (cumulative Gaussian). The 50% cut-off point of the psychometric curve (i.e. the point at which there are equal amounts of left-side-higher and right-side higher responses for a given condition) was taken as the PSE. We then subtracted the pre-test PSEs from the post-test PSEs of each condition to obtain the size of the adaptation after-effect (taking the direction of the adaptation slant into account).

#### Exclusion of participants from the analysis

We removed all participants who needed more than 40 trials to finish at least one of the staircases in the design, since this is indicative of the staircases not converging. This resulted in the removal of 2 female participants. This means that 11 participants (9 female, age range: 19–38 years) remained for the analysis.

### Results experiment 1

**Fig 2:**
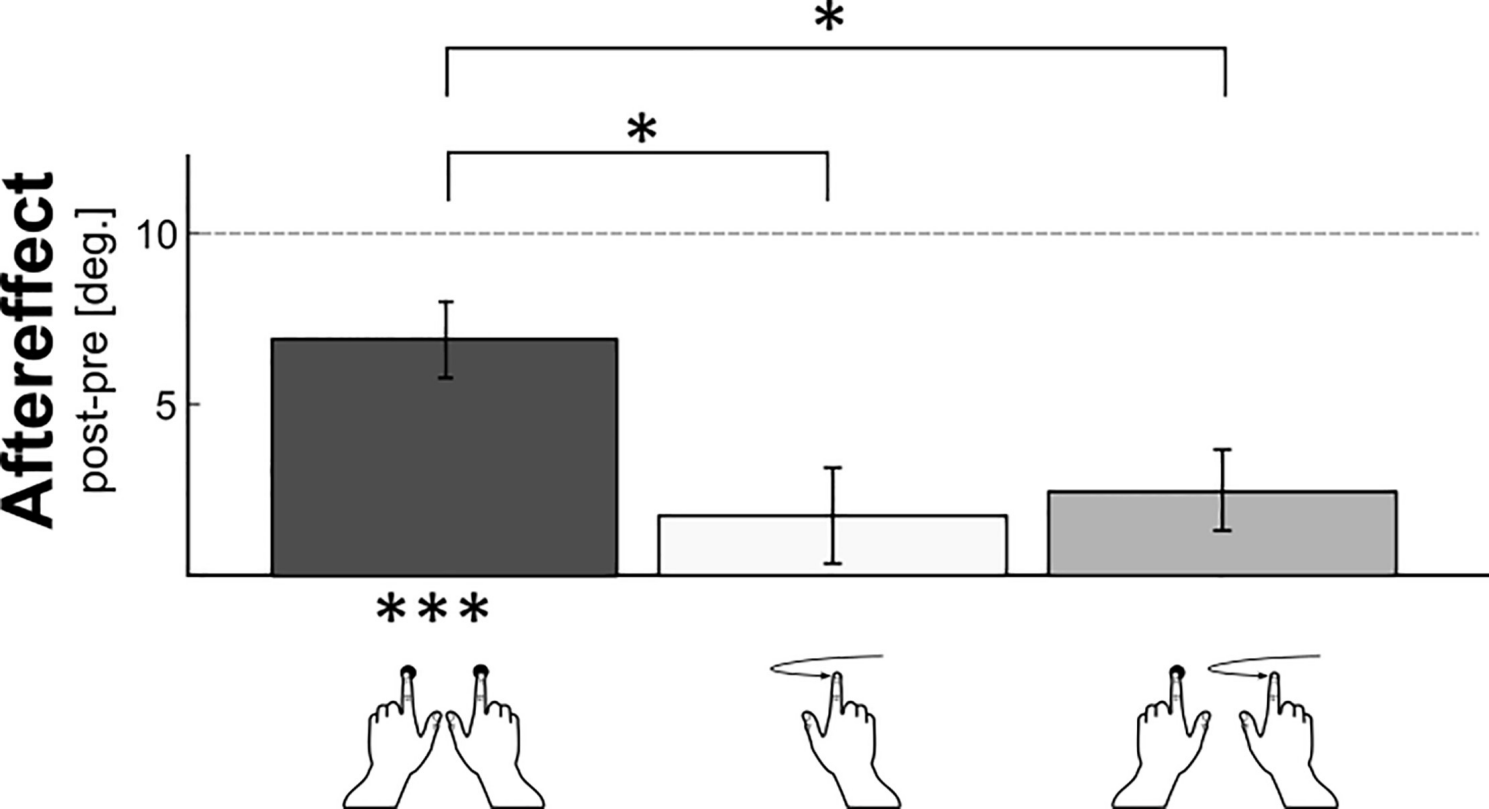
Adaptation aftereffect and transfer of bimanual static adaptation. On the x-axis the different movement conditions are shown: Static Bimanual (left), the Dynamic Unimanual (middle) and the Mixed Exploration condition (right). The y-axis shows the aftereffects as calculated by subtracting the PSE of the pre-test from the PSE of the post-test. The dashed line indicates the point at which full adaptation would occur. Error bars represent the standard error.

After the Static Bimanual adaptation to a 10.0 deg surface slant, there was a significant aftereffect (Fig 2) when using the Static Bimanual exploration mode also in the test phases (two-tailed One sample t-test against 0, t(10) = 6.00, p<0.001; Bonferroni corrected using an alpha of 0.0167; Cohen’s d = 1.81), though adaptation was not complete (6.9 deg ± 1.1 deg instead of the 10.0 deg adaptation angle). This means that the angle at which the surface was perceived as level had significantly changed between pre- and post-test.

However, there was no significant transfer of adaptation to the Dynamic Unimanual exploration mode, (One sample t-test against 0, t(10) = 1.22, p = 0.25; Bonferroni corrected using an alpha of 0.0167; Cohen’s d = 0.37). There was also no significant transfer to the Mixed Bimanual condition, in which a mixture of the static and the dynamic exploration was used (One sample t-test against 0, t(10) = 2.14, p = 0.06; Bonferroni corrected using an alpha of 0.0167; Cohen’s d = 0.64). Using an one-way ANOVA we tested for differences between the conditions and found a significant effect (F(2,30) = 5.14, p = 0.01; partial η^2^ = 0.26). Post-hoc paired-samples t-tests revealed—after Bonferroni correction using an alpha of 0.0167—that the size of the aftereffect in the Static Bimanual condition differed significantly from the Mixed Bimanual condition (Paired t-test, t(10) = 3.20, p<0.01; Cohen’s d = 0.96) as well as the effect for the Dynamic Unimanual condition (Paired t-test, t(10) = 3.18, p<0.01; Cohen’s d = 0.96). The aftereffects for the Mixed Bimanual condition and the Dynamic Unimanual condition, however, were not significantly different from each other (Paired t-test, t(10) = 0.57, p = 0.58; Cohen’s d = 0.17). Together these results indicate that bimanual haptic slant adaptation is possible if the information of the two hands is non-redundant and furthermore, that this adaptation is condition specific.

### Discussion experiment 1

In Experiment 1, we tested if bimanual adaptation is possible and if this adaptation transfers to a dynamic movement condition when using only one hand. Our results show a significant aftereffect when the two index fingers statically touch the adaptation surface (Static Bimanual condition). This shows that also with slant input derived from two hands adaptation is possible (Bimanual Adaptation).

Since in our experiment a slant-estimate for Static Bimanual exploration was only possible when the information of both index fingers is combined, it seems that the interaction between the hands is adaptable. However, it has to be noted that this adaptation cannot occur at the same level at which intermanual transfer was previously observed for dynamic exploration [2, 6], since in the present Experiment 1 the adaptation did not transfer to exploration modes that involved a dynamic component. This is in line with a study by Van Dam et al. [3], which showed that information from unimanual static and dynamic exploration modes do not transfer between modes even when using the same hand. Van Dam et al., concluded that static haptic adaptation is largely a low-level, i.e. posture based adaptation, which is dependent on the exploration mode. Our results of Experiment 1 are consistent with this conclusion. They show that it is enough to include a dynamic component in the mode of surface exploration to decrease adaptational transfer effects. This can be seen most clearly in the Mixed Bimanual condition in which the position estimates of the two hands are both available and informative about the slant, yet no transfer to this condition was observed. One explanation for this might be an independent adaptation of static and dynamic exploration, as found by Van Dam et al. [3], even in the case of bimanual exploration. Since the exploration mode used during adaptation was the Bimanual Static mode, the neurons/receptors coding for static exploration adapted, but the neurons coding for dynamic exploration did not adapt. Thus, the dynamic exploration is unaffected by static adaptation aftereffects.

This, however, raises the question whether a distal stimulus, i.e. a haptic slant, is needed to adapt to slant. From the study by Van Dam et al. [3] it is known that static unimanual haptic adaptation to slant is heavily dependent on the hand posture. If this is also the case for bimanual adaptation a distal stimulus should not be necessary for adaptation to occur. Thus, we conducted a second experiment in which in one condition participants adapted to a haptically rendered surface and in a second condition to just the finger positions by holding the index fingers at fixed points in the air. For pure adaptation of posture, touching an actual object and thus receiving haptic feedback from the object should not be necessary. In other words, removing the object and adapting purely proprioceptively by holding the fingers in mid-air should elicit the same effect as adapting by touching an actual surface.

## Experiment 2

The results of Experiment 1 showed that bimanual slant adaptation is exploration mode specific and no transfer was found to exploration modes that included a dynamic component. This suggests that even static bimanual adaptation may be heavily posture based. If so, this raises the question whether an object is really needed for haptic slant adaptation to occur. To investigate this, we conducted a second experiment in the present study. This second experiment included two conditions: In the first condition, we adapted participants in a static bimanual fashion (i.e. keeping static contact with the surface using both index fingers) to a surface slant that was rendered haptically (surface present). That is, like in the first experiment the surface could be felt and haptic feedback was provided when touching it. In the second condition, participants adapted–also in a static bimanual fashion–to just the corresponding position in space. That is, in the second condition participants held their fingers in mid-air at the positions where the slant was programmed, just that now there was no surface that could be felt (surface absent). Should aftereffects be present in the condition without any haptic feedback and furthermore, should those effects transfer to the condition in which haptic feedback is available and vice versa, this would be clear evidence that the static bimanual adaptation is posture based. However, if there are no aftereffects in the condition without haptic feedback, or should the aftereffects not transfer, this would point towards adaptation needing the interaction with a physical surface rather than being purely posture based. Several studies showed that for instance Area 2 of the primary somatosensory cortex is particularly sensitive to the specific combinations of proprioceptive (posture) and tactile (haptic feedback) information (e.g. [21–23]). This would suggest that also the combination of posture and haptic force feedback (and thus the presence of a surface) could play an important role in haptic shape perception in general and adaptation in particular.

### Material and methods experiment 2

#### Participants

A total of 14 people volunteered to participate in the experiment (9 female, age range: 20–32 years). They were all self-reported right-handed and received 6€/h as compensation for participation. They gave informed consent prior to the experiment.

#### Setup & conditions

Because we were interested in the object dependence of slant adaptation, we had two conditions: adaptation to slant when a surface provided haptic feedback (Surface Present condition) and adaptation to “slant” by holding the fingers in mid-air without touching a surface (Surface Absent condition). The setup was the same as in Experiment 1. In Experiment 2, however, we used only the Static Bimanual exploration mode for both adaptation as well as testing. The experiment was divided into two sessions, which for each participant were performed on two different days. In one session, the participants adapted in the Surface Present condition and in the other they adapted to posture alone in the Surface Absent condition. The order of the sessions was counterbalanced across participants. In both sessions, the test conditions were the Surface Present and the Surface Absent conditions, to test for condition specific adaptation as well as transfer.

#### Procedure

**Fig 3:**
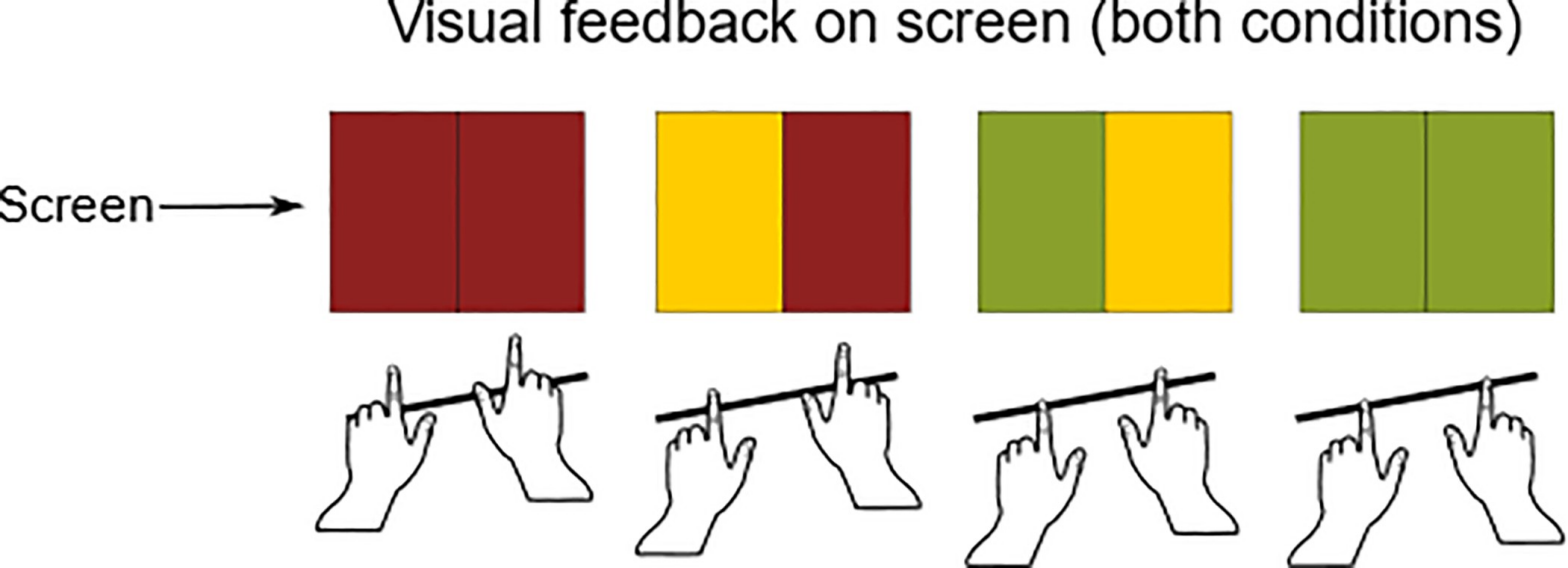
Presenting information about the vertical finger distance relative to the surface. The computer screen was split in half. The left side corresponded to the left finger, the right side to the right finger. The solid line represents the surface, i.e. a touchable surface in the Surface Present condition and in the Surface Absent condition an imaginary surface. Participants initially moved their hand downward, i.e. along the gravitational axis, to reach the correct position for a given trial. The colour of each screen half depended on how close the participant's fingers were to the surface: the corresponding screen half turned from red to yellow 15 mm above and below the surface and when the participant (would) touch the surface the corresponding screen half turned green (2.5 mm above the surface for the surface present condition, 5 mm above and below the surface for the surface absent condition).

The same adaptation procedure as in Experiment 1 was used. This time, however, no information about the upcoming trial was given. Instead the screen gave information about the finger position relative to the surface (see Fig 3). This was particularly important for the Surface Absent condition because the participant could not feel the surface. Yet we needed them to take up the specific postures that relate to a given surface slant. For providing the participant with information about the distance of the finger to the surface, the screen was split in half. The right half of the screen corresponded to the right finger and the left half of the screen to the left finger. To inform the participant about the vertical position of the finger, a traffic light symbolism was used. If the screen-half was red the finger(s) were far away from the surface. As soon as the finger was closer than 15 mm to the surface, the corresponding screen half turned yellow and as soon as the finger was closer than 2.5 mm (Surface Present) or 5 mm (Surface Absent) the corresponding screen half turned green. The two thresholds for the green light for the Surface Present and Surface Absent conditions were different because we observed in pilot experiments that with a 5 mm threshold in the Surface Present condition the participants sometimes did not touch the surface at all during a trial if their approach was too careful. On the other hand, for the Surface Absent condition the 2.5 mm threshold turned out to be too difficult to maintain in mid-air for both fingers simultaneously. For this reason, we chose to use two slightly different thresholds in the two conditions. Depending on the condition, the participants could feel a surface (Surface Present) or not (Surface Absent). When both fingers were in the “green zone” the trial time started. After one second the screen turned black and the participant decided which side was higher using the response zones as in Experiment 1 (see Fig 1B). Then the next trial started.

The same statistical analysis as for Experiment 1 was used and Bonferroni correction was applied for the one- and paired-sample t-tests to correct for multiple comparisons (i.e. alpha was set to 0.0125).

### Results experiment 2

**Fig 4:**
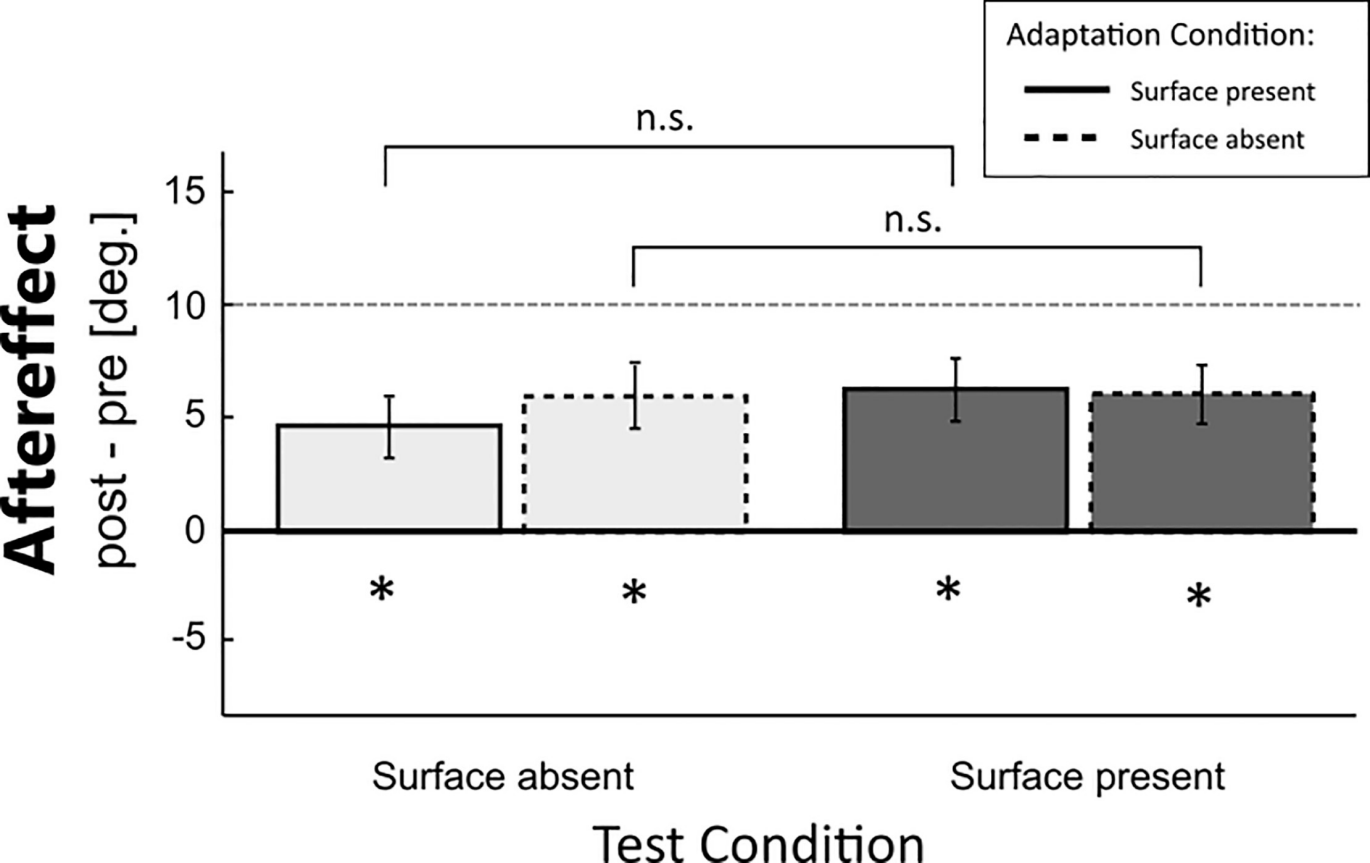
Adaptation effects in the two main conditions. Solid outline: The adapted condition was the Surface Present condition; Dashed outline: The adapted condition was the Surface Absent condition. On the x-axis the two test conditions are shown. The y-axis shows the adaptation aftereffect. The dashed line marks the point at which full adaptation would occur. The error bars represent the standard error.

When adapting using the Surface Present condition (Fig 4, bars with solid outline), the adaptation after- and transfer effects for the test conditions Surface Present (5.8 deg ± 1.6 deg) and Surface Absent (4.6 deg ± 1.4 deg) were both significantly different from zero (Surface Present, One-sample t-test: t(13) = 3.66, p<0.01; Cohen’s d = 0.98; Surface Absent, One-sample t-test: t(13) = 3.18, p<0.01; Cohen’s d = 0.85) and not significantly different from each other (Paired t-test: t(13) = 1.02, p = 0.33; Cohen’s d = 0.27). These results confirm the finding from Experiment 1 that bimanual adaptation to surface slant using the two index fingers in a non-redundant static fashion, leads to adaptation aftereffects for test-conditions that have the same static exploration mode. Experiment 2 shows that this is true regardless of the presence of the surface. The bars in Fig 4 with a dashed outline show the results when the participants adapted to the Surface Absent condition. In this case participants held their fingers in mid-air at the indicated positions using the screen traffic light system. Similar to the results for adapting with a rendered surface (solid outline bars), the adaptation aftereffect of the Surface Absent test condition (4.6 deg ± 1.4 deg) is significantly different from zero (One-sample t-test: t(13) = 3.15, p<0.01; Cohen’s d = 0.84). Again this aftereffect fully transferred to the Surface Present test condition (5.1deg ± 1.3deg) which was also significantly different from zero (One-sample t-test: t(13) = 3.89, p<0.01; Cohen’s d = 1.04). Again, there was no significant difference between the two test conditions (t(13) = 0.42, p = 0.68; Cohen’s d = 0.11).

The fact that the Surface Absent and Surface Present conditions led to similar aftereffects and that these fully transferred between conditions, clearly demonstrates that posture and not object presence is a crucial factor in slant adaptation. However, this raises the question of whether we are dealing with bimanual adaptation at all. That is, it is not clear whether it is the relative static posture between the hands that adapts (i.e. the way the position of one hand may in part be judged in relation to the other hand), or if the results of Experiment 1 and 2 can fully be explained by very low-level unimanual posture adaptation (each hand adapting in isolation but to slightly different postures and in this way leading to the observed aftereffects). If it is the relative positions between the hands that adapts this relative difference, and thus the adaptation aftereffect, should fully transfer when testing at a different height compared to where adaptation occurred. Adapting one hand only by keeping it in a certain posture for a period of time should however in this case not lead to any “slant” aftereffects, since no adaptation of relative hand positions should occur. In contrast, in the case of pure unimanual posture adaptation, proprioceptors and muscles in each hand and arm get adapted. This should then lead to slightly misperceived position estimates when the hand is moved away from the adaptation position (e.g. through muscle conditioning; for further information see e.g. [15, 16, 24–26]). This means that it should be possible to find adaptation effects when adapting only a single hand to a certain height and then testing how this affects position estimates when the hand is next moved to a different height. If both hands adapt at the same time in this manner but to slightly different positions, this can account for the results in the previous experiments.

### Material and methods experiment 3

To distinguish between an effect due to a relative static posture adaptation and an effect based on a low-level unimanual adaptation, we conducted a third experiment. Here the assumption was the following: if adaptation is based on the position of each hand (unimanual) rather than the relative position between the hands, a change in position, here height, after adaptation should lead to an overestimation of the change in height for the adapted hand(s) [16, 25, 26]. However, if the relative position between the hands gets adapted, i.e. the difference in positions between the left hand and the right hand adapts over time rather than each hand adapting individually, a change in height should not show an overestimation of the height change when adapting unimanually. Rather in this case, even after bimanual adaptation, aftereffects for the relative position between the hands should not depend on the test height at all and thus remain equal at different testing heights. To test these different predictions Experiment 3 included adaptation conditions that involved both hands set at a “slant” by placing the two hands at different heights corresponding to that “slant”. Moreover, Experiment 3 included conditions in which only one hand was adapted by placing it at a specific height for a period of time. For both types of adaptation, the test condition consisted of placing one hand at one of three pre-defined heights and setting the other hand such that it was perceived to be at the same height.

#### Participants

For Experiment 3 ethical approval was obtained from the University of Essex Ethics Committee. A total of 11 people, including the authors CG and LD volunteered to participate in the experiment (10 female, age range: 20–40 years). They were all self-reported right-handed and student volunteers received course credits as compensation for their participation. They gave informed consent prior to taking part in the experiment.

#### General setup

The findings that the observed “slant” aftereffects seem to be posture based, rather than requiring haptic force feedback about the object, allowed us to move away from the PHANToM force feedback devices which have only a limited workspace. For Experiment 3 we instead used the Oculus Rift VR headset and touch controllers (Oculus Rift CV1 Facebook Technologies, LCC) to both guide the participants to the correct hand position for each adaptation and test condition as well as measure the hand positions using the touch controllers. This furthermore allowed us to measure adaptation aftereffects at more extreme heights compared to what would be possible with the PHANToM force feedback devices. To be able to verify that the participants followed the instructions, the hand positions during various stages of the trials were recorded with a sampling frequency of 90 Hz.

**Fig 5:**
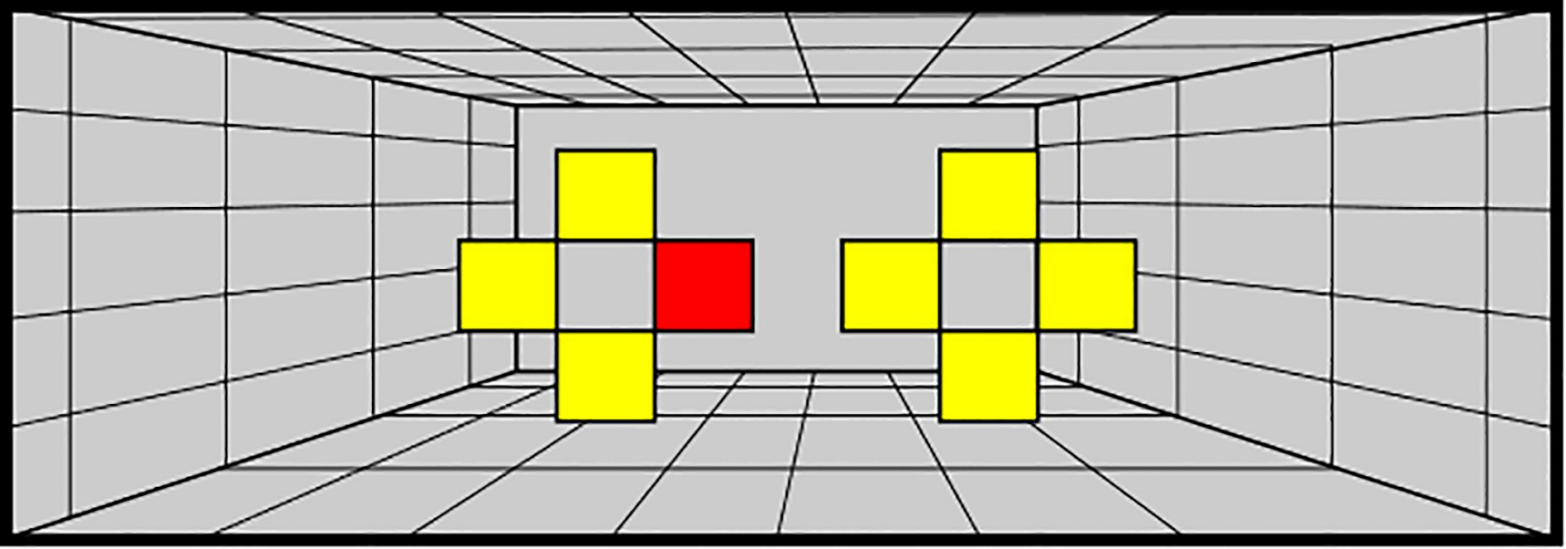
Visual feedback the participants received to get to the correct positions with their hands. Shown is an example for a bimanual adaptation phase. In this example the participant holds the right hand in the correct x- and y-coordinates (+/- 1.0 cm). The left hand is held at the correct y-coordinates (+/- 1.0 cm) but more than 1.0 cm to the right of the goal coordinates. Therefore, the right square of the left cross is shown red.

In Experiment 3, in the pre- and post-test phases the participants were guided to place one of their hands at a certain position in 3D space using a visual guidance system in the VR headset (see Fig 5). Once their hand was in the correct position, they then had the task to match the height of their “set hand” with their “free hand”. This way we obtained on each individual trial a measure of the height differences at which the participants perceived their two hands to be at the same level. During the adaptation phase, the same visual guidance system was used to have participants place either one or both of their hands (depending on the condition) in such predefined 3D positions.

To guide the participants to the correct position for the set hand(s), we gave visual feedback as seen in Fig 5. The left cross corresponds to the left hand, the right cross to the right hand. The goal for the participant was to get all squares yellow. As soon as the controller left the goal area in a certain direction, the corresponding square(s) turned red indicating to the participant they had to place their hand more in the opposite direction. The goal area was defined as a 3-dimensional box spanning 2.0 cm in the horizontal and vertical directions and 4.0 cm in depth. The goal area along the depth direction was double the size since it was harder to maintain compared with the other two dimensions. Furthermore, the depth direction was not of main interest in this experiment and therefore did not require the same level of precision. To control for the right position in depth, we used vibration. As soon as the participant moved out of the goal area to the front or back the controller(s) started to vibrate, telling the participant to correct for depth. It is important to note that the visual placement of the crosses was fixed for the whole course of the experiment and thus its position in virtual space did not correspond in any meaningful way to the position of the hand in real space. Therefore, this guidance system only provided feedback to correct the hand position if necessary and did not provide visual feedback as to the precise 3D coordinates of the hand(s) in space. Note that the cross(es) for the “set hand” in the visual display remained visible throughout the experiment (i.e., also during adaptation and test phases) in order to allow readjustments in case participants unintentionally left the goal area with their hand.

For bimanual adaptation both crosses of the visual guidance system were shown. The goal areas for the hands were 7.0 cm to the left of the body midline for the left hand (using the position of the VR-headset as a reference) and 7.0 cm to the right for the right hand, with a height difference between the hands of 10.0 cm centred around the shoulder area (20.0 cm below the VR-headset). The hands furthermore needed to be placed at a distance in depth of 30.0 cm. Note that the height difference roughly corresponds to a slant of 36 deg instead of 10 deg as used in the previous experiments. This was done since we had to allow for the range of goal areas in which participants placed their hands as well as for the idea that we were working with hand position rather than fingertip positions. A “slant” of 10 deg would have easily been lost in the possible variable placement of the hands within the respective goal areas.

For unimanual adaptation only the cross corresponding to the adapted hand was shown using the colour representations described above. The adapting position would again be placed 7.0 cm to the left or right, depending on whether the left or right hand was adapted, at roughly shoulder height (i.e. 20.0 cm below the position of the VR headset) and 30.0 cm in depth from the VR headset. The squares making up the cross corresponding to the non-adapting hand were visible but black. The non-adapting hand was held down in a relaxed fashion.

#### General procedure

The experiment started with a short training block in which the participants were familiarized with the setup and how to interpret the colour coding and vibrational feedback. After the training session the experiment started. The experiment was done in a blocked design, i.e. each adaptation condition was done in a separate block of trials. After each block there was a break of 10 minutes in which the participants were allowed to rest their arms, take off the VR headset and were encouraged to do things with their hands to help the de-adaptation (e.g. drink, eat a snack, using the smartphone etc.). After the break, the next block with the next adaptation condition started.

Each block consisted of a pre-test phase, the adaptation phase and a post-test phase, as in the previous experiments.

#### Bimanual adaptation condition

**Fig 6:**
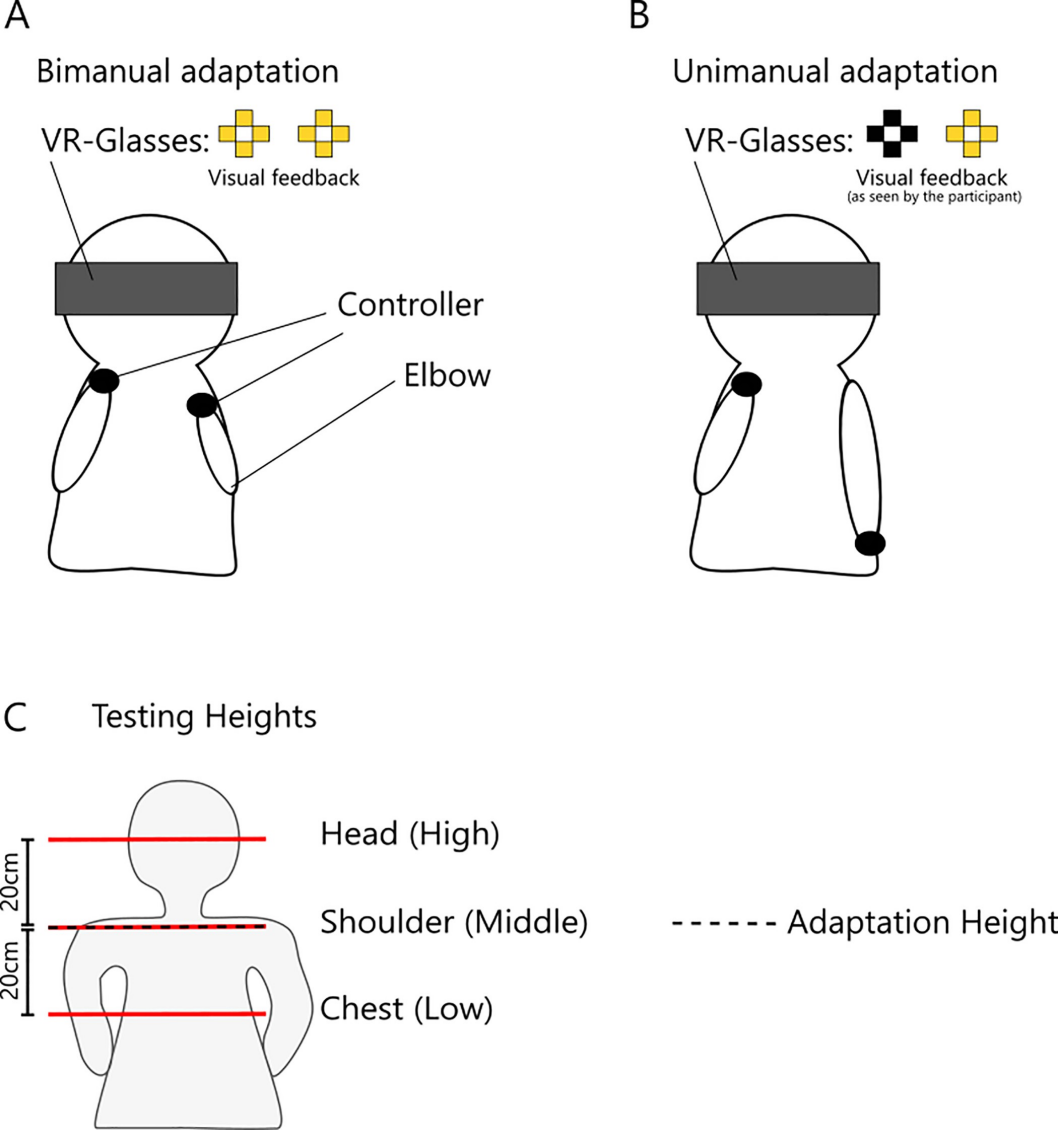
Sketches of the conditions and testing heights in the third experiment. A: Controller positions during bimanual adaptation. Both hands are raised to keep the two crosses in the visual feedback yellow; B: Controller positions during unimanual adaptation (right hand). The adapted hand is raised (in this example the right hand) whereas the left hand is held in a relaxed position. In the unimanual conditions the cross corresponding to the unadapted hand was shown black. Note that in the picture the visual feedback shown is the one the participant sees in the VR glasses (i.e. mirrored to the observer); C: Testing heights of the experiment. The red lines show the testing heights in relation to the participant’s body. Note that we used the coordinates of the VR headset as the reference for the correct placement of the set hand. Thus, the testing positions relative to the body differed slightly between participants, depending how tall the participant was. The dashed line marks the adaptation height.

To be able to compare our results of Experiment 3 to the previous experiments we had a bimanual adaptation condition in which both hands had a goal area during the adaptation phases. Each participant performed two blocks of trials for the bimanual adaptation condition. In one block the right hand was held higher during the adaptation phases (positive slant), in the other block the left hand was held higher during adaptation (negative slant). Fig 6 shows sketches of the different adaptation conditions and the different testing heights. In Fig 6A the controller positions (for a positive slant) as well as the visual feedback given by the VR glasses are shown. For the main adaptation phase participants held their hands in the indicated goal area for 30 seconds. In the pre- and post-test phases, we used the testing conditions as explained above: one hand (the set hand) was guided to one of the three testing heights (see Fig 6C) using the visual guidance system (the other cross was black) and participants next had to match it with the other hand (the free hand) without any visual feedback. Once satisfied that their hands were at the same height, participants pressed either “X” or “A” on one of the controllers to start the next trial. Which hand was used as the set hand and which as the free hand was counterbalanced across trials. Per set hand each testing height was repeated three times. This led to a total number of 36 test trials for each block (2 hands x 3 heights x 3 repetitions = 18 test trials for each of the pre and post-test phases). The order of the conditions was randomized in each test-phase.

As in the previous experiments, the post-test differed from the pre-test, i.e. that each test-trial was preceded by a 4 second top-up adaptation interval in which participants were guided to take up the same hand positions as during the main adaptation phase. Participants were notified what they needed to do at each stage through messages displayed in the virtual environment (e.g. keep hands in the same position for adaptation intervals, or move the “free” hand to the same height as the “set” hand in the test-phases).

#### Unimanual adaptation condition

In the unimanual adaptation condition, only one hand was adapted at shoulder height. There were two blocks of trials for the unimanual condition. In one block the left hand was adapted, in the other block the right hand was the adapted hand. For the adaptation phases the hand to be adapted was guided to the correct adaptation height using the visual guidance system explained above. Participants were instructed to hold the other arm down in a resting position during the main adaptation phase (30 seconds) as well as during the top-up adaptation intervals (4 seconds) of the post-test phase. Fig 6 shows the controller positions and the visual feedback for a right-hand adaptation condition. Note that in this case one cross, namely the cross of the unadapted hand, was shown in black, i.e. no visual feedback was provided for the non-adapting hand. For test trials the adapting hand for that block was guided to one of the three testing heights as seen in Fig 6C and participants next had to try and match the felt height with their non-adapted hand. Each test-height was repeated 3 times in each of the pre- and post-test phases. Therefore, the number of trials in the unimanual adaptation conditions was 18 trials per block (9 trials in the pre-test + 9 trials in the post-test).

As indicated above, we used three different testing heights in the pre-test phase as well as in the different post-test phases to which one hand (the “set hand”) of the participant was guided to. One testing height was at eye level (called “Head”), as determined by the location of the VR headset in space. The second testing height was at 20.0 cm below the centre of the VR headset which roughly corresponded to shoulder height (called “Shoulder”). The third height for testing was at 40.0 cm below the centre of the VR headset, which roughly corresponds to chest height (called “Chest”, see Fig 6C).

#### Analysis

To analyse the effects of adaptation, we analysed the height hand settings for pre- and post-test trials. This is the height at which participants felt their hands to be at the same height. To determine these heights, we took the y-coordinate of the hands at the moment the participant pressed the “X” or “A” button on the Oculus Touch^TM^ controllers to indicate that the matching of the hands was complete. We then subtracted the coordinate of the left and right hand to calculate the relative height difference for each trial. Furthermore, we pooled the data across the two blocks for each of the bimanual and unimanual adaptation conditions (mirroring the data where necessary), as the effects were symmetric for the two hands. Here handedness did not play a role. For the statistical analysis we compared the mean results in terms of the relative height differences in the settings for each adaptation condition and each testing height to zero with a one-sample t-test and we used paired-sample t-tests for comparisons between the different testing heights for each adaptation condition. Bonferroni correction was applied for the one- and paired-sample t-tests to correct for multiple comparisons (i.e. alpha was set to 0.0167).

### Results experiment 3

**Fig 7:**
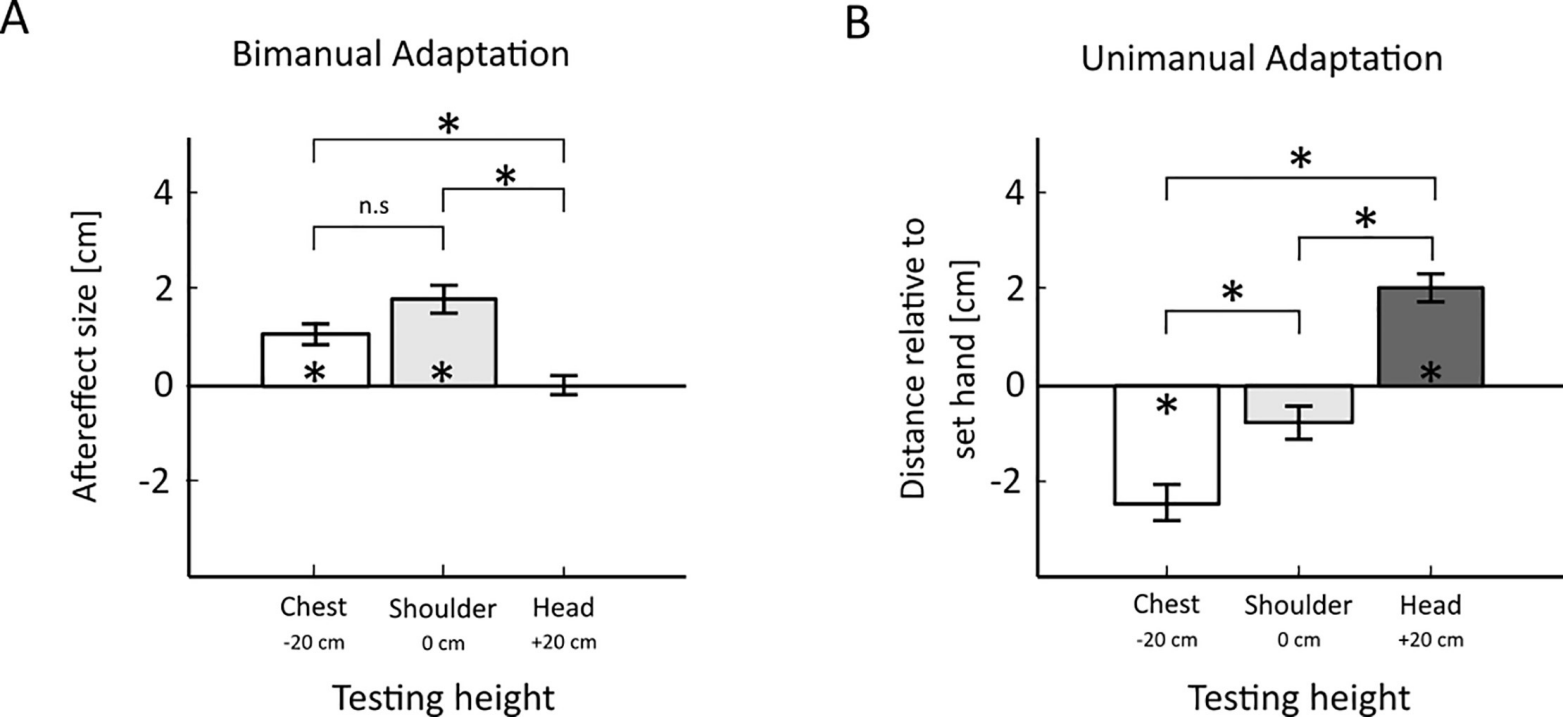
Results of the bimanual and unimanual adaptation. A: Results of the Bimanual Adaptation conditions; B: Results of the Unimanual Adaptation conditions. The x-axes show the different testing heights relative to the adaptation height. The y-axis in A shows the bimanual “slant” aftereffect and in the unimanual adaptation the height of the “free” hand relative to the set hand. “Shoulder” is the adaptation height, “Chest” is the testing height 20 cm below the adaptation height and “Head” is the testing height 20 cm above the adaptation height. The errorbars represent standard errors.

Fig 7 shows the results for the Bimanual Adaptation condition of Experiment 3 (Fig 7A) together with the Unimanual Adaptation condition (Fig 7B). The x-axis shows the height at which the test was performed relative to the height that was used for adaptation and the y-axis the size of the aftereffect in cm.

Using these results, we first verified whether the same effects of bimanual adaptation also appear with the VR setup, i.e. in 3D virtual space without force feedback. To do so here in Exp. 3 we used the one bimanual adaptation condition that was the most similar to the static bimanual adaptation conditions of the previous Experiments 1 and 2, except for using the Oculus Rift with the Touch controllers instead of the PHANToM force-feedback devices. This was the bimanual adaptation condition for which both test and adaptation occurred at the same “Shoulder” level height (see Fig 7A, the middle bar). It can be seen that an aftereffect occurs also in this case (one-sample t-test against zero for “Shoulder” level: t(10) = 5.06, p < 0.01; Cohen’s d = 1.53) despite the fact that there were no boundaries and thus no force or other kind of external haptic feedback was present.

Next, we tested for effects of adaptation transfer at “Chest” and “Head” level also in the Bimanual Adaptation condition (Fig 7A, “Chest” level: left bar and “Head” level: right bar). It can be seen that such a transfer effect occurred at least to some extent for the “Chest” level (one sample t-test: t(10) = 4.22, p<0.01; Cohen’s d = 1.27) but not for the “Head” level (one sample t-test: t(10) = 0.01, p = 0.99; Cohen’s d<0.01). However, both the results for the “Shoulder” and “Chest” level are significantly different to the “Head” level (paired samples t-test Chest-Head: t(10) = 5.36; p<0.001; Cohen’s d = 1.62; Shoulder-Head: t(10) = 4.47; p<0.01; Cohen’s d = 1.35) but not significantly different to each other (Chest-Shoulder: t(10) = 1.46; p = 0.18; Cohen’s d = 0.44). Thus, the transfer effects, when testing at different heights than the adapted height, were significantly reduced only in one condition (“Head” level) whereas a significant transfer effect was observed for the second transfer condition (“Chest” level). Since these results are mixed, it is difficult to make any strong conclusions. However, the above shown results—together with the results of the previous experiments (which point towards receptor based adaptation)—hint towards the assumption that it may not be the relative position between the hands at a bimanual stage that gets adapted, in which case we would have expected the adaptation aftereffect to more or less fully transfer to both the different testing heights. Since this is not the case, adaptation may perhaps actually be occurring at the unimanual level.

We used the Unimanual Adaptation condition to verify this suggestion. If bimanual adaptation occurs at the unimanual level, adapting only one hand to a certain height and then moving it to another height, should lead to an overshoot in the position estimation of this hand. Thus, in the Unimanual Adaptation condition only one hand was adapted to the “Shoulder” level and we then measured whether aftereffects, i.e. a misjudgement of the “set hands” position, occurred at the same and different testing heights. The results are shown in Fig 7B. The x-axis shows the testing height relative to the adaptation height (“Chest” = -20.0 cm, “Shoulder” = 0.0 cm, “Head” = +20.0 cm); the y-axis represents the height difference between the free hand and the adapted “set” hand at which the hands are perceived to be at the same height. When testing at the same height as the adaptation took place, no significant difference in height perception occurred (one-sample t-test t(10) = 1.85, p = 0.09; Cohen’s d = 0.56. The results show a significantly negative distance for the testing height at “Chest” level, indicating that the participants perceived the adapted hand to be lower than it actually was (t(10) = 5.34, p<0.001; Cohen’s d = 1.61). For the testing height “Head” however, the distance is significantly positive, indicating that the participants perceived the adapted hand to be held at a higher position than it actually was (t(10) = 7.14, p<0.001; Cohen’s d = 2.15). This means that for both the “Chest” testing level and the “Head” testing level the participants overestimated the distance that the hand had moved from the adaptation level, which is consistent with adaptation effects in perception. Furthermore, the results of the three conditions are significantly different from each other (paired-sample t-test Chest-Shoulder: t(10) = 3.32, p<0.01; Cohen’s d = 1.00; Chest-Head: t(10) = 7.86, p<0.001; Cohen’s d = 2.37; Shoulder-Head: t(10) = 7.00, p<0.001; Cohen’s d = 2.11). These results confirm that haptic adaptation can occur for a single hand position individually.

### Discussion experiment 3

The results again confirm that bimanual adaptation in 3D space is possible without needing to touch any surface. This means, that even when the participant is simply holding their hands in a certain position in 3D space without external force feedback, adaptation aftereffects occur. The results of the unimanual adaptation show that the participants significantly misjudge the position of the adapted hand when this hand is moved. That is, the adapted hand is perceived significantly lower when moved downwards and significantly higher when moved upwards. This effect was already described by Gregory et al. [26] and was confirmed here. Furthermore, this shows that adaptation to height is possible with a single hand and thus points towards adaptation at the level of the individual hands (e.g. through adaptation of the muscle spindles) rather than an adaptation of the two hands in relation to each other. Though the results for the bimanual condition are not entirely conclusive, the finding that the Bimanual Adaptation transfer effect is significantly reduced when tested at “Head” level is in line with this interpretation. Adaptation of relative hand positions instead of adaptation of each individual hand should be independent of the location/posture at which adaptation and testing occurs, and we would expect aftereffects to fully transfer to any other location. In the present experiment this would mean that for bimanual adaptation the results at non-adapted locations (“Chest” and “Head” levels) should have been the same as at the adapted height (“Shoulder” level). This is evidently not the case in the present results when testing at “Head” level. This absence of transfer of the aftereffect to “Head” level cannot simply be due to biomechanical constraints because we did find strong unimanual aftereffects at this height. Therefore, our results show that at the very least such an adaptation is again posture dependent and does not necessarily transfer to all non-adapted postures. It has to be noted however, that since we did not observe a significant reduction of adaptation transfer when testing at the “Chest” level, it would be premature to completely rule out a role of adaptation of relative hand positions.

Taken together, the results from all three experiments confirm that the posture at which adaptation occurs is the most important factor. This indicates at the very least a very important role for unimanual adaptation processes for generating such aftereffects. Moreover, the unimanual condition in Experiment 3 highlights that bimanual aftereffects could potentially even be fully explained by unimanual adaptation.

Lastly, it is of interest to note that, across the three experiments we observed very similar adaptation aftereffects for the bimanual adaptation conditions. Yet, in Experiment 3 we used controllers, which had to be grasped by the participants while in the other experiments we used the PHANToM robot arms in which only the fingertips were used. Combined, the present results therefore suggest that the haptic slant adaptation is likely related to the position of the arms and shoulders and not solely on the finger positions per se.

## General discussion

In the first part of the present study, we investigated if bimanual adaptation to slant is possible in conditions in which it is essential that the information from both hands is used (non-redundant information). The results of Experiment 1 showed that Static Bimanual slant adaptation does occur. Furthermore, the Static Bimanual adaptation aftereffect transferred neither to the Dynamic Unimanual condition nor to the Mixed Bimanual condition in which dynamic and static exploration were mixed and position information for both fingers was available (Mixed Bimanual). These results extend the findings by Van Dam and colleagues [3], who found that static and dynamic exploration adapt independently when tested within one hand, to the bimanual case. In Experiment 2 we tested whether a distal stimulus is needed for adaptation and showed that a physical object is not necessary to elicit haptic adaptation aftereffects. This suggests that also bimanual adaptation is posture based. Finally, Experiment 3 provides evidence that this adaptation is most likely linked to adaptation at the level of the individual hands rather than at a level at which the relative position differences between the hands is recalibrated.

### Bimanual adaptation to slant

In the present study we showed, for the first time, that adaptation to a haptic feature, in this case slant, also works when the two hands are simultaneously involved in the adaptation process. In earlier studies adaptation to haptic features was already shown (size and volume: e.g. [27]; curvature: e.g. [2, 4–6]; slant: [3]), but only within one hand. Our study extends these findings by showing that adaptation to slant also occurs when slant is estimated using two fingers from different hands. Here it is important to note that in this study as well as in the study on slant adaptation by Van Dam et al. [3], one static finger was not enough to estimate the slant of the surface. One needs a second finger to be able to make a judgment of the surface slant by estimating the difference in position between the fingers. In the present study the two fingers used were from the two different hands and thus the slant could only be estimated by combining information from the two hands. Our findings show that this nevertheless resulted in adaptation aftereffects.

### No transfer of aftereffects between exploration modes

In this study we furthermore showed that the bimanual slant adaptation is exploration mode specific and does not transfer to conditions with a dynamic exploration component. Estimating slant is also possible by using a single finger and moving it in a dynamic fashion to sample the height differences over time by sliding over the surface. Thus, there are two ways to obtain information about slant (statically and dynamically) that intuitively might share common neural pathways since they serve the same purpose. In this case, the adaptation should be independent of the exploration mode and transfer between them. The Static Bimanual adaptation found in this study, however, did not transfer to conditions that had any form of dynamic component, even with two hands present on the surface and thus relative position estimates between the hands still being available (Mixed Bimanual Condition of Experiment 1). An explanation for the lack of transfer is that Static Bimanual adaptation is dependent on the exploration mode–i.e. based on the postures of the individual hands (for a review see [15, 16])–rather than at a stage at which both hands are represented. At first glance, this seems to contradict the findings of Van der Horst et al. [2, 6], who showed that adaptation to curvature transfers from the adapted hand to the non-adapted hand. Intermanual transfer was particularly found for dynamic information gathering, which points towards a bimanual processing stage [2]. However, van der Horst and colleagues [6] also found that intermanual transfer was much reduced or absent when using static contact with the curvature, showing that the bimanual processing stage may be very particular to dynamic exploration only. This is in line with an independence between static and dynamic exploration modes and, rather than Static Bimanual adaptation occurring at a bimanual level, suggested an alternative explanation for the present results of Experiment 1. In the present case, the slant percept is likely derived by estimating the distances between the fingers along the horizontal and vertical dimensions. If the perceived positions of the individual fingers adapt (rather than the slant), this would lead to changes in slant perception after adaptation, despite the adaptation not specifically occurring at a bimanual processing stage that estimates the slant. This would also explain why we did not find transfer to the Mixed Bimanual condition, since in that case one finger is not providing a stable position estimate. Yet, moving the fingers can provide a, perhaps more accurate, estimate of the slant based on its dynamic exploration that has remained unadapted. This is consistent with the results by Van Dam et al. [3] who showed that adaptation does not transfer between dynamic and static exploration with the same hand.

All in all, our results strongly suggest that Static Bimanual exploration is processed differently compared to the bimanual stage for the dynamic exploration mode that Van der Horst and colleagues [2] proposed. Furthermore, the results of both the current experiment 1 and 2 suggest the strong posture dependence found by Van Dam and colleagues [3] is also true for bimanual static adaptation to slant. This is in line with a study by Vogels et al. [5], that showed that for unimanual adaptation posture has an effect on the adaptation aftereffect. In their study participants had to make either a fist, hold the hand passive in mid-air or bend and stretch the fingers after adaptation and before testing. They then tested how fast the curvature adaptation decays in the different conditions. They found that when a fist was made before testing, the decay time is significantly shorter than when holding the hand passive in mid-air. That is, the fist posture of the hand interfered with the adaptation aftereffect. This showed that posture is a factor in haptic adaptation, which is in line with our findings. However, the study by Vogels and colleagues [5] did not investigate the bimanual case nor whether there is a difference between adapting to posture alone and posture plus haptic feedback from the touched object (or own hand).

### Influence of cutaneous cues

Due to the fact that we used force-feedback devices to present the slanted surface, there were no direct cutaneous cues present for the slant of the surface. Instead, the cues available in the present study were the force-feedback from the surface (Experiments 1 and 2) and proprioceptive cues about the hand/finger postures (all three experiments). This is different from most previous studies in which real objects were presented and for which thus both proprioceptive and cutaneous cues were available. From previous research it is known that such cutaneous cues also adapt when available (e.g. [28]). However, even despite the difference in the presence of cutaneous cues the results from this study are very consistent with the work from Vogels et al. [4, 5, 29] and Van der Horst et al. [2, 6], which are all studies involving real objects and thus included both proprioception and cutaneous cues. Hence, it is likely–at least for adaptation to global shape–that cutaneous cues play only a minor role. This may however be very different for adaptation to predominantly tactile stimuli, such as the texture of a surface or other stimuli that fit within the area of a single fingertip, for which adaptive interactions between the hands have been observed in the CNS to at least some degree [13, 14].

### Posture-based haptic slant adaptation

Experiment 2 addressed whether haptic adaptation is a purely proprioceptive adaptation. If static adaptation is indeed posture based, after-effects should be found even in the absence of a physical surface during adaptation. In Experiment 2, we therefore removed the haptic surface during adaptation in one condition and the results show that Static Bimanual adaptation indeed also occurs when adapting to posture alone (i.e. with the fingers held in mid-air). Furthermore, there are no differences in magnitude of adaptation between the Surface Present and Surface Absent conditions and adaptation fully transfers between these two conditions. This indicates that haptic feedback, i.e. the increased force when touching the surface and the differences in muscle tension induced by this, makes no difference for haptic slant adaptation. This strongly supports the idea by Van Dam et al. [3], that static haptic adaptation to slant is mainly posture based. In the study by Van Dam and colleagues [3] hand posture was a crucial factor for finding aftereffects in adaptation when testing using static contact with the object. They found that the average hand posture during the dynamic adaptation phases had a strong impact on the transfer effect to a static testing condition and a testing condition in which posture and dynamic components were combined. This leads to the assumption that static haptic slant adaptation is rather a proprioceptive adaptation that does not rely on haptic feedback from an object, at least not to any measurable extent. Our results are consistent with the idea that each finger adapts individually to its own posture based on the proprioceptive sensory input from, for instance, muscle spindles and skin stretch (for reviews see e.g. [15, 16]). For adapting one hand, posture adaptation makes sense, given that it can be linked to one group of muscles and joints. Interestingly, for adapting to slant using two hands, where the fingers of the separate hands act independently without a mechanical link, we still found similar results, despite the hands needing to share information to estimate slant.

### Comparison between possible explanations for the site of adaptation

Based on the finding that proprioceptive posture is a key factor for bimanual adaptation it seems plausible that the proprioceptors of the individual hands are involved in the adaptation process. In theory adaptation at this level could fully explain the present findings. However, it is important to note that there is an alternative explanation for the present results, which is that adaptation occurs at a higher level at which the position of one hand is compared to the position of the other hand. When estimating the “slant”, or as in the Surface Absent condition of our second experiment the relative positions of the two fingers, this requires the information of the two hands to be shared. This means that this comparison necessarily has to take place at a processing stage at which both hands are represented. Adaptation at such a stage, rather than adaptation at the level of the individual hands, would for instance explain why the adaptation surface itself tends to feel more level as time progresses. In other words, each hand may adapt to the position of the opposite hand which then would lead to the stable percept of a level surface over time. This is in line with the idea that symmetry is preferred by the body (e.g. for vision: [30, 31]; for locomotion: [32]; for hand movements: [33, 34]; for joint information processing: [35]). In the case of adaptation to slant one hand or finger is higher than the other, possibly driving the adaptation to a point at which both hands/fingers feel level. Thinking of natural statistics this makes sense. If the two arms are passively hanging down from our shoulders, the fingers, hands and arms are roughly in symmetry. This raises the idea that during adaptation the brain is adjusting what symmetry between the limbs feels like. In other words: a reference for the position of one hand could in fact be the other hand, i.e. the right hand's position is the reference for the left hand's position and vice versa. This way one would adapt in a way that the perceived distance between the two hands decreases. This would also lead to the alignment aftereffects found in the present study.

Since the two theories are in conflict with each other, we conducted a third experiment in which we tested whether unimanual adaptation to a certain height leads to adaptation aftereffects. If the adaptation from the previous experiments was based on muscle spindle and skin stretch adaptation, it should be possible to find adaptation effects when adapting only one hand. If the previously found effects were based on adaptation of the relative hand positions at a bimanual stage, unimanual adaptation should not show any effects. The results show that when adapting one hand to a certain position and then moving the hand up or down, leads to the impression that the hand moved further than it actually did. This is in line with the findings of Gregory et al. [26] who found that when flexing or stretching the elbow flexors the perceived limb position changes. The reason for this is that the firing rate of the involved receptors in the muscles and joints decrease their background discharge rates over time when held static in a certain position. Thus, when moving again the firing rate of the receptors in relation to the background discharge rate is higher, leading to the impression that a larger distance was moved [16, 36]. The findings of the third experiment match these previous findings, therefore suggesting that each arm or hand adapts individually. Since the task for the participants was to match the height of the adapted hand with the unadapted hand, the brain still needs to compare the position of the two hands. However, since adaptation leads to the misjudgement of the position of the adapted hand [25], also the height difference at which the hands are perceived as level is misjudged. As shown in Experiment 3, the effects of unimanual adaptation were quite strong and therefore likely dominated also when adapting bimanually to slant. This in part, if not completely, can also explain the findings for the bimanual adaptation conditions in this experiment if the shift in perceived position depends on the distance moved. It has to be noted though that the conditions in Experiment 3 did not allow us to work out the extent to which unimanual adaptation alone can account for all the adaptation effects in this study. Therefore, a role of adaptation at a bimanual comparison stage, though unlikely, cannot yet be completely ruled out. However, based on the present findings it can be safely assumed that if such adaptation at a bimanual comparison stage exists its role is likely relatively minor.

## Conclusion

Our results show that it is possible to adapt bimanually to slant using static touch and that this adaptation does not transfer to conditions that involve a dynamic exploration component, even if the relative positions of both hands are still informative about the slant. Furthermore, we demonstrated that for haptic adaptation the presence of an object is not necessary to elicit adaptation aftereffects and that the observed aftereffects are based on the adaptation of posture for each hand and arm individually. Hence, taken together we conclude that although slant estimation needs the input of both hands, Static Bimanual adaptation is largely of proprioceptive nature at the level of the individual hands. That is, the posture information of the individual hands is already biased before it arrives at the stage in the CNS at which the hand positions are compared.

## Supporting information

S1 File Video conditions experiment 3. This video shows the different conditions in experiment 3 as seen by the participant through the VR glasses.
(MP4)
